# Adenosine A_2A_R blockade prevents neuroinflammation-induced death of retinal ganglion cells caused by elevated pressure

**DOI:** 10.1186/s12974-015-0333-5

**Published:** 2015-06-10

**Authors:** Maria H. Madeira, Filipe Elvas, Raquel Boia, Francisco Q. Gonçalves, Rodrigo A. Cunha, António Francisco Ambrósio, Ana Raquel Santiago

**Affiliations:** Institute for Biomedical Imaging and Life Sciences (IBILI), Faculty of Medicine, University of Coimbra, 3004-548 Coimbra, Portugal; CNC.IBILI, University of Coimbra, 3004-517 Coimbra, Portugal; Association for Innovation and Biomedical Research on Light (AIBILI), 3000-548 Coimbra, Portugal; CNC-Center for Neuroscience and Cell Biology, University of Coimbra, 3004-517 Coimbra, Portugal; Faculty of Medicine, University of Coimbra, 3000-548 Coimbra, Portugal; IBILI, Faculty of Medicine, University of Coimbra, Azinhaga de Santa Comba, 3004-548 Coimbra, Portugal

**Keywords:** Microglia, Adenosine, Neuroprotection, Glaucoma

## Abstract

**Background:**

Elevated intraocular pressure (IOP) is a major risk factor for glaucoma, a degenerative disease characterized by the loss of retinal ganglion cells (RGCs). There is clinical and experimental evidence that neuroinflammation is involved in the pathogenesis of glaucoma. Since the blockade of adenosine A_2A_ receptor (A_2A_R) confers robust neuroprotection and controls microglia reactivity in the brain, we now investigated the ability of A_2A_R blockade to control the reactivity of microglia and neuroinflammation as well as RGC loss in retinal organotypic cultures exposed to elevated hydrostatic pressure (EHP) or lipopolysaccharide (LPS).

**Methods:**

Retinal organotypic cultures were either incubated with LPS (3 μg/mL), to elicit a pro-inflammatory response, or exposed to EHP (+70 mmHg), to mimic increased IOP, for 4 or 24 h, in the presence or absence of the A_2A_R antagonist SCH 58261 (50 nM). A_2A_R expression, microglial reactivity and neuroinflammatory response were evaluated by immunohistochemistry, quantitative PCR (qPCR) and enzyme-linked immunosorbent assay (ELISA). RGC loss was assessed by immunohistochemistry. In order to investigate the contribution of pro-inflammatory mediators to RGC loss, the organotypic retinal cultures were incubated with rabbit anti-tumour necrosis factor (TNF) (2 μg/mL) and goat anti-interleukin-1β (IL-1β) (1 μg/mL) antibodies.

**Results:**

We report that the A_2A_R antagonist (SCH 58261) prevented microglia reactivity, increase in pro-inflammatory mediators as well as RGC loss upon exposure to either LPS or EHP. Additionally, neutralization of TNF and IL-1β prevented RGC loss induced by LPS or EHP.

**Conclusions:**

This work demonstrates that A_2A_R blockade confers neuroprotection to RGCs by controlling microglia-mediated retinal neuroinflammation and prompts the hypothesis that A_2A_R antagonists may be a novel therapeutic option to manage glaucomatous disorders.

## Background

Glaucoma is the third leading cause of visual impairment and the second cause of blindness worldwide [1]. It is defined as a group of chronic degenerative optic neuropathies, characterized by the irreversible and progressive loss of retinal ganglion cells (RGCs) and damage of the optic nerve (RGC axons). Although glaucoma is a multifactorial disease, elevated intraocular pressure (IOP) is a major risk factor and the current treatments are mainly focused on reducing IOP [2]. However, many patients continue to lose vision despite the control of IOP, and neuroprotective strategies aimed to prevent RGC loss are necessary [3].

Increasing evidence has shown that neuroinflammation has an important role in the pathogenesis of glaucoma [4–6]. Accordingly, microglial cells display an activated amoeboid-like morphology at the early stages of glaucoma [7–10]. In parallel, there is an increased expression and release of pro-inflammatory cytokines [e.g. tumour necrosis factor (TNF), interleukin-1β (IL-1β)] and nitric oxide (NO) in the glaucomatous eye [11–14]. The importance of this microglia-associated neuroinflammation in glaucoma is underscored by the observation that the control of microglia activation [15–17] or of pro-inflammatory cytokine expression [4, 18] can prevent the loss of RGC in animal models of glaucoma.

Microglia-associated neuroinflammation is also involved in different brain disorders [19]. Adenosine is a neuromodulator, which can control inflammatory reactions [20, 21] and microglia reactivity [22–24] mainly through the activation of its G-protein-coupled receptor of the A_2A_ receptor (A_2A_R) subtype [25]. Accordingly, A_2A_R antagonists afford robust neuroprotection upon ischemia, epilepsy or Alzheimer’s or Parkinson’s disease [25].

All these evidence prompt the hypothesis that A_2A_R antagonists may also control the microglia-associated neuroinflammation and loss of RGC in animal models of glaucoma. Therefore, the main aim of this work was to investigate whether A_2A_R blockade modulates retinal microglia reactivity, neuroinflammation and loss of RGC triggered by lipopolysaccharide (LPS) or elevated hydrostatic pressure (EHP).

## Materials and methods

### Animals

Adult Wistar rats were housed in certified local facilities, in a temperature- and humidity-controlled environment, and were provided with standard rodent diet and water ad libitum, under a 12 h light/12 h dark cycle. All procedures involving animals were approved by the Ethical Committee of the Faculty of Medicine of the University of Coimbra/Center for Neuroscience and Cell Biology and are in agreement with the Association for Research in Vision and Ophthalmology statement for animal use.

### Organotypic retinal cultures

Wistar rats (8–10 weeks old) were euthanized and their eyes enucleated. Retinas were dissected in a Ca^2+^- and Mg^2+^-free Hank’s balanced salt solution (HBSS (in mM) 137 NaCl, 5.4 KCl, 0.45 KH_2_PO_4_, 0.34 Na_2_HPO_4_, 4 NaHCO_3_, 5 glucose; pH 7.4) and placed in tissue culture inserts (Millipore; 0.4-μm pore diameter) with the ganglion cell layer (GCL) facing up. The retinas were cultured for 4 days in DMEM-F12 with GlutaMAX I, supplemented with 10 % heat-inactivated foetal bovine serum and 0.1 % gentamicin (all from Life Technologies) at 37 °C, in 5 % CO_2_ humidified atmosphere, as previously described [26]. The culture medium was replaced at culture days 1 and 2.

### Culture treatments

Organotypic retinal cultures were either incubated with LPS (3 μg/mL, Sigma-Aldrich) or exposed to EHP (70 mmHg above atmospheric pressure) for 4 or 24 h. The concentration of LPS was chosen amongst three tested concentrations of LPS (1, 3 and 5 μg/mL), as the lowest triggering an increase in iNOS immunoreactivity in the majority of microglial cells in organotypic retinal cultures. For the EHP experiments, we used a custom-made humidified pressure chamber equipped with a pressure gauge and a pressure regulator, which allowed maintaining a constant pressure with an air mixture of 95 % air and 5 % CO_2_, as described previously [27]. The chamber was placed in an oven at 37 °C. The magnitude of pressure elevation (70 mmHg above atmospheric pressure) was chosen in accordance with previous studies [27, 28]. For ambient pressure experiments, the organotypic retinal cultures were kept in a standard 5 % CO_2_ humidified incubator.

The cultures were incubated with a selective A_2A_R antagonist (SCH 58261; Tocris Bioscience), tested at a selective and supra-maximal concentration (50 nM) [24], which was added 45 min before exposure to LPS or EHP. To test the role of extracellular adenosine, organotypic cultures were treated with 1 U/mL adenosine deaminase (ADA; Roche Applied Science) which catalyzes the irreversible deamination of adenosine to inosine. In order to investigate the contribution of pro-inflammatory mediators to RGC loss, the organotypic retinal cultures were incubated with rabbit anti-TNF (2 μg/mL; Peprotech) and goat anti-IL-1β (1 μg/mL; R&D Systems) antibodies, or with corresponding immunoglobulin Gs (IgGs), 45 min before exposure to LPS or EHP for 24 h. Organotypic cultures were also incubated with 20 ng/mL TNF and 10 ng/mL IL-1β (ImmunoTools) to evaluate if TNF and IL-1β, by themselves, lead to RGC loss.

### Immunohistochemistry

Organotypic cultures were washed with phosphate-buffered saline (PBS (in mM) 137 NaCl, 2.7 KCl, 10 Na_2_HPO_4_ and 1.8 KH_2_PO_4_; pH 7.4) and fixed with ice-cold ethanol for 10 min at 4 °C. After washing in PBS, cultures were blocked and permeabilized with 10 % normal goat serum, 3 % bovine serum albumin and 0.1 % Triton X-100 in PBS, for 1 h, and then incubated with the primary antibody (Table 1) for 48 h at 4 °C. After washing, cultures were incubated overnight with the secondary antibody (Table 1), at 4 °C. Retina cultures were then washed and incubated with 4′,6-diamidino-2-phenylindole (DAPI; 1:1000) for 15 min, to stain nuclei. After washing, the preparations were flat-mounted on slides and coverslipped using Glycergel mounting medium.

**Table 1:** Primary and secondary antibodies used in immunohistochemistry

	Supplier	Host	Dilution
Primary antibodies			
Anti-A_2A_R	Santa Cruz Biotechnology	Goat	1:100
Anti-CD11b	AbD Serotec	Mouse	1:250
Anti-iNOS	BD Biosciences	Rabbit	1:200
Anti-Brn3a	Chemicon	Mouse	1:500
Secondary antibodies			
Alexa Fluor anti-mouse 568	Life Technologies	Donkey	1:200
Alexa Fluor anti-goat 488	Life Technologies	Rabbit	1:200
Alexa Fluor anti-rabbit 488	Life Technologies	Goat	1:200

### Image acquisition and densitometric analysis

The preparations were observed with a confocal microscope (LSM 710, Zeiss) on an Axio Observer Z1 microscope using an EC Plan-Neofluar 40x/1.30 Oil DIC M27 objective, and, from each quadrant, at least three images of the GCL were randomly acquired (encompassing central and peripheral retina), in a total of 12 images. The settings and exposure times were kept identical for all conditions within each experiment. Densitometric analysis was performed using the public domain ImageJ program (http://rsb.info.nih.gov/ij/). Corrected total cell fluorescence (CTCF) was calculated as previously described [29] using the following formula:

$$ \mathrm{CTCF} = \mathrm{Integrated}\ \mathrm{density}-\left(\mathrm{area}\ \mathrm{of}\ \mathrm{selected}\ \mathrm{cell}\kern0.37em \times \mathrm{mean}\ \mathrm{fluorescence}\ \mathrm{of}\ \mathrm{background}\ \mathrm{reading}\right) $$

### Circularity index and skeleton analysis

Morphological alterations of microglia were estimated as previously described [30] using the confocal images of the retinal organotypic cultures labelled with anti-CD11b. Briefly, the particle measurement feature of ImageJ was used to automatically evaluate the circularity index (CI) of microglia, using the formula CI = 4*π*(area/perimeter^2^). A circularity index of 1.0 indicates a perfect circle.

The microglial cell complexity and branch length were assessed by skeleton analysis using ImageJ software, as described previously [31]. Briefly, confocal images were converted to 8-bit format, followed by noise de-speckling to eliminate single-pixel background fluorescence. Then, images were converted to binary images, which were analyzed using AnalyzeSkeleton plugin (http://fiji.sc/AnalyzeSkeleton) to assess the number of microglial cell processes, number of branch endpoints and maximum branch length for each cell. These results were analyzed as average per frame.

### ATP quantification

The extracellular levels of adenosine triphosphate (ATP) were quantified with a luciferase ATP bioluminescence assay kit (Sigma-Aldrich) as we previously described [32]. Briefly, the supernatants were collected and immediately stored at −80 °C until used. Then, 80 μL of these supernatant were added to a white 96-well plate (designed for bioluminescence) placed in a VICTOR multilabel plate reader (PerkinElmer). The luciferin-luciferase ATP assay mix (40 μL) was automatically loaded in each well, and the luminescence output was converted to ATP concentration by interpolation of a standard curve, which was linear between 2 × 10^−12^ and 8 × 10^−5^ M. ATP concentration was normalized to the total amount of protein of each retina, which was determined by the bicinchoninic acid assay (Pierce Biotechnology).

### NO production assay

The production of NO was quantified by the Griess reaction method in the supernatants of the culture medium. The culture medium was centrifuged (10,000*g* for 10 min) and the supernatant stored at −80 °C until use. Then, the supernatant was incubated (1:1) with Griess reagent mixture (1 % sulfanilamide in 5 % phosphoric acid with 0.1 % *N*-1-naphthylethylenediamine) for 30 min at room temperature and in the dark. The optical density was measured at 550 nm using a microplate reader (Synergy HT; Biotek). The nitrite concentration was determined from a sodium nitrite standard curve.

### Quantitative real-time PCR

Total RNA was extracted using Qiagen RNeasy Mini Kit (Qiagen), according to the instructions provided by the manufacturer. The concentration and purity of total RNA were determined using NanoDrop ND1000 (Thermo Scientifics). Then, 1 μg of total RNA was reversed transcribed using a NZY First-Strand cDNA Synthesis Kit according to the manufacturer instructions (NZYTech, Portugal). The resultant complementary DNA (cDNA) was treated with RNase-H for 20 min at 37 °C, and a 1:2 dilution was prepared for quantitative PCR (qPCR) analysis. All cDNA samples were stored at −20 °C until further analysis.

Genomic DNA contamination was assessed with a conventional PCR for β-actin using intron-spanning primers (Table 2), as described previously [33]. SYBR Green-based real-time qPCR was performed using a StepOnePlus PCR system (Applied Biosystems). The PCR conditions were as follows: iTaq™ Universal SYBR® Green Supermix (Bio-Rad), 200 nM primers (Table 2) and 2 μL of 1:2 dilution of cDNA, in a total volume of 20 μL. Cycling conditions were a melting step at 95 °C for 15 s, annealing elongation at 60 °C for 45 s and extension at 72 °C, with 40 cycles. A dissociation curve at the end of the PCR run was performed by ramping the temperature of the sample from 60 to 95 °C, while continuously collecting fluorescence data. Ct values were converted to *relative quantification* using the 2^−ΔΔCt^ method [34]. Three candidate housekeeping genes (*hprt*, *Ywhaz* and *GAPDH*) were evaluated using NormFinder, a Microsoft Excel Add-in [35], and *hprt* was the most stable gene throughout all experimental conditions and samples and, therefore, was used as the housekeeping gene.

**Table 2:** Primers used in qPCR and RT-PCR

Gene	GenBank number	Forward	Reverse	Amplicon size (bp)
Adora2A	NM_053294	5′ - GGCTATCTCTGACCAACA - 3′	3′ - TGGCTTGACATCTCTAATCT - 5′	106
TNF	NM_012675	5′ - CCCAATCTGTGTCCTTCT - 3′	3′ - TTCTGAGCATCGTAGTTGT - 5′	90
IL-1β	NM_031512	5′ - ATAGAAGTCAAGACCAAAGTG - 3′	3′ - GACCATTGCTGTTTCCTAG - 5′	109
Nos II	NM_012611	5′ - AGAGACAGAAGTGCGATC - 3′	3′ - AGAGATTCAGTAGTCCACAATA - 5′	96
hprt	NM_012583	5′ - ATGGGAGGCCATCACATTGT- 3′	3′ - ATGTAATCCAGCAGGTCAGCAA - 5′	76
actb	NM_031144	5′ - GCTCCTCCTGAGCGCAAG - 3′	3′ - CATCTGCTGGAAGGTGGACA - 5′	75

### Enzyme-linked immunosorbent assay

Culture media was centrifuged (10,000*g* for 10 min) and the supernatant was collected and stored at −80 °C until use. The levels of TNF and IL-1β in the culture supernatants were quantified by enzyme-linked immunosorbent assay (ELISA), according to the instructions provided by the manufacturer (Peprotech).

### Retinal ganglion cell counting

Retinal ganglion cells were identified by immunohistochemistry staining with an antibody anti-Brn3a (RGC marker), and confocal images of the GCL were acquired (as described above). The number of Brn3a-immunoreactive cells per image was counted using ImageJ Cell Counter plugin (http://rsbweb.nih.gov/ij/plugins/cell-counter.html). Results represent the average of Brn3a-immunoreactive cells per image.

### Statistical analysis

The results are presented as mean ± standard error of the mean (SEM). The data were analyzed using the non-parametric Kruskal-Wallis test, followed by Dunn’s multiple comparison test, as indicated in the figure legends. The statistical analysis was performed using the Prism 6.0 software for Mac OS X (GraphPad Software, Inc).

## Results

The retinal organotypic culture is particularly useful to evaluate molecular and cellular mechanisms in the retina because the retinal structure is maintained [26]. Thus, we used this experimental model to investigate the ability of A_2A_R to control neuroinflammation and RGC death triggered by LPS or EHP (to mimic an increase in IOP).

### LPS and EHP increased the expression of A_2A_R in retinal microglial cells in the GCL

Since the A_2A_R modulation system undergoes a gain of function upon noxious brain conditions [25], we first assessed if this also occurred in the retina. Therefore, we investigated if LPS or EHP up-regulated the expression of A_2A_R and bolstered the source of adenosine responsible for the activation of A_2A_R, i.e. ATP-derived adenosine [36].

**Fig. 1:**
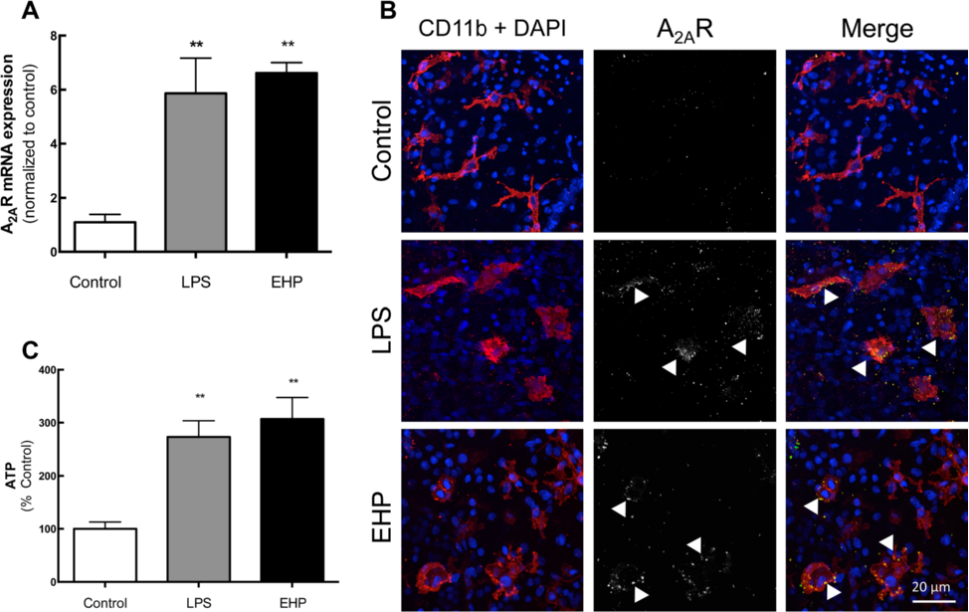
LPS or EHP increases A_2A_R expression and density in retinal microglia and increase the extracellular ATP levels. Retinal organotypic cultures were challenged with LPS (3 μg/mL) or EHP (+70 mmHg) for 24 h. **a** A_2A_R mRNA expression was assayed by qPCR. Results are presented as fold change of the control, from six to ten independent experiments. **b** Organotypic retinal cultures were immunostained for A_2A_R (*grey*/*green*; *arrowheads*) and CD11b (microglia marker; *red*) and imaged in the GCL using a confocal microscope. Nuclei were stained with DAPI (*blue*). Representative images obtained from four independent experiments. **c** The extracellular levels of ATP in the medium were quantified by luciferin-luciferase ATP-dependent reaction. Results are expressed as percentage of control and are mean ± SEM of six to eight independent experiments. **P < 0.01, different from control; Kruskal-Wallis test, followed by Dunn’s multiple comparison test

LPS or EHP exposure for 4 h significantly increased A_2A_R messenger RNA (mRNA) expression in the retina by 5.3- and 6.0-fold (*n* = 6–10), respectively (Fig. 1a). Accordingly, 4 h after exposure to LPS or EHP, A_2A_R immunoreactivity increased mainly in CD11b-positive cells in the GCL (Fig. 1b), indicating that A_2A_R in the GCL are mainly present in microglia.

Extracellular ATP levels in control conditions were 0.6 ± 0.3 pmol/μg protein (*n* = 8) and significantly increased by 173.8 ± 30 and 215.1 ± 40 % after 24 h of exposure to LPS or EHP (*n* = 6–8), respectively (Fig. 1c).

### A_2A_R blockade prevented the alterations of microglia morphology triggered by LPS or EHP

**Fig. 2:**
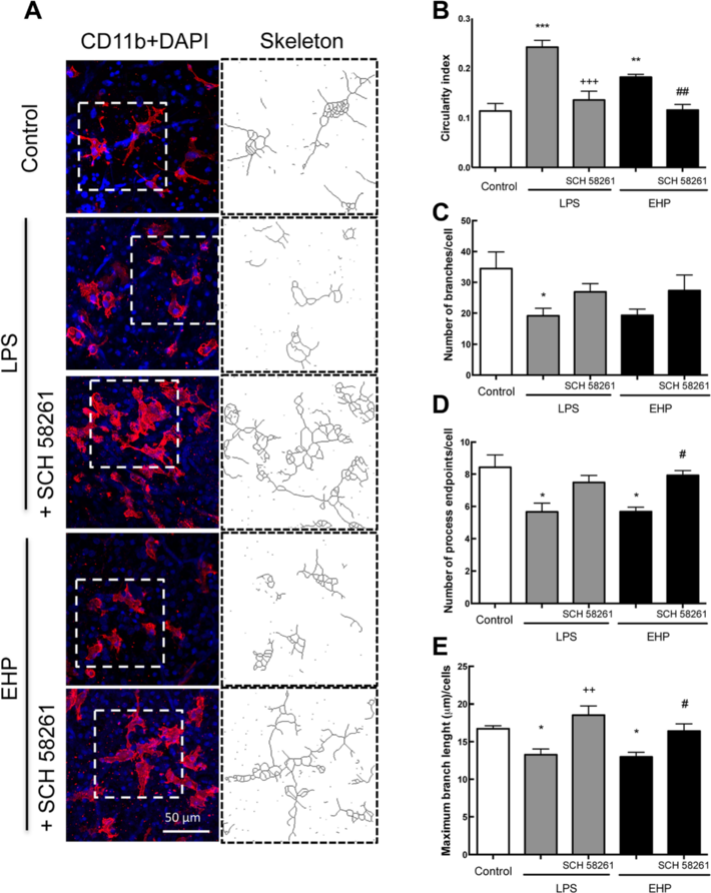
Blockade of A_2A_R prevents microglia morphological changes induced by LPS or EHP. Retinal organotypic cultures were pretreated with the A_2A_R antagonist SCH 58261 (50 nM) and then challenged with LPS (3 μg/mL) or EHP (+70 mmHg) for 4 h. **a** Organotypic retinal cultures were immunostained for CD11b (microglia marker; *red*) and imaged in the GCL using a confocal microscope. Nuclei were stained with DAPI (*blue*). Representative images obtained from four to five independent experiments. **b** the circularity index, **c** number of branches per cell, **d** number of process endpoints per cell and **e** the maximum branch length (μm) per cell were calculated for the different experimental conditions. The *bar graphs* present data as mean ± SEM of four to five independent experiments. *P < 0.05, **P < 0.01 and ***P < 0.001, different from control; ^++^ *P* < 0.01 and ^+++^ *P* < 0.001, different from LPS; ^#^ *P* < 0.05 and ^##^ *P* < 0.01, different from EHP; Kruskal-Wallis test, followed by Dunn’s multiple comparison test

Modification of cell morphology is one of the hallmarks of microglia activation and has been widely used to categorize different activation states [19]. As shown in Fig. 2a, under control conditions, microglial cells (i.e. CD11b-positive cells) in the GCL typically presented a ramified morphology [circularity index (CI) 0.110 ± 0.02, *n* = 7; Fig. 2b], compatible with a *surveying* phenotype. After 24 h of exposure to LPS or EHP, microglia morphology changed to a more amoeboid-like morphology (CI 0.242 ± 0.014 and 0.182 ± 0.006, respectively; *n* = 5–8, *P* < 0.05 vs. control). Incubation with the selective antagonist of A_2A_R (SCH 58261, 50 nM) prevented the LPS- and EHP-induced alterations of microglia circularity index (*n* = 5–8) (Fig. 2b). In addition, skeleton morphological analysis was used to further document more subtle morphological changes compatible with microglial activation. Retinal microglia from LPS- and EHP-treated cultures presented a decrease in the number of branches (Fig. 2c), endpoints (Fig. 2d) and maximum branch length (Fig. 2e) compared to the control condition. The blockade of A_2A_R prevented these alterations, indicating that A_2A_R blockade blunted LPS- and EHP-induced microglia reactivity.

#### Blockade of A_2A_R prevented microglia production of NO

Since the activation of microglial cells leads to the production of pro-inflammatory and cytotoxic factors like NO both in vivo and in vitro [37], we tested if A_2A_R could control the up-regulation of inducible nitric oxide synthase (iNOS), which plays a critical role in neuroinflammation by generating high amounts of NO in reactive microglia [38].

**Fig. 3:**
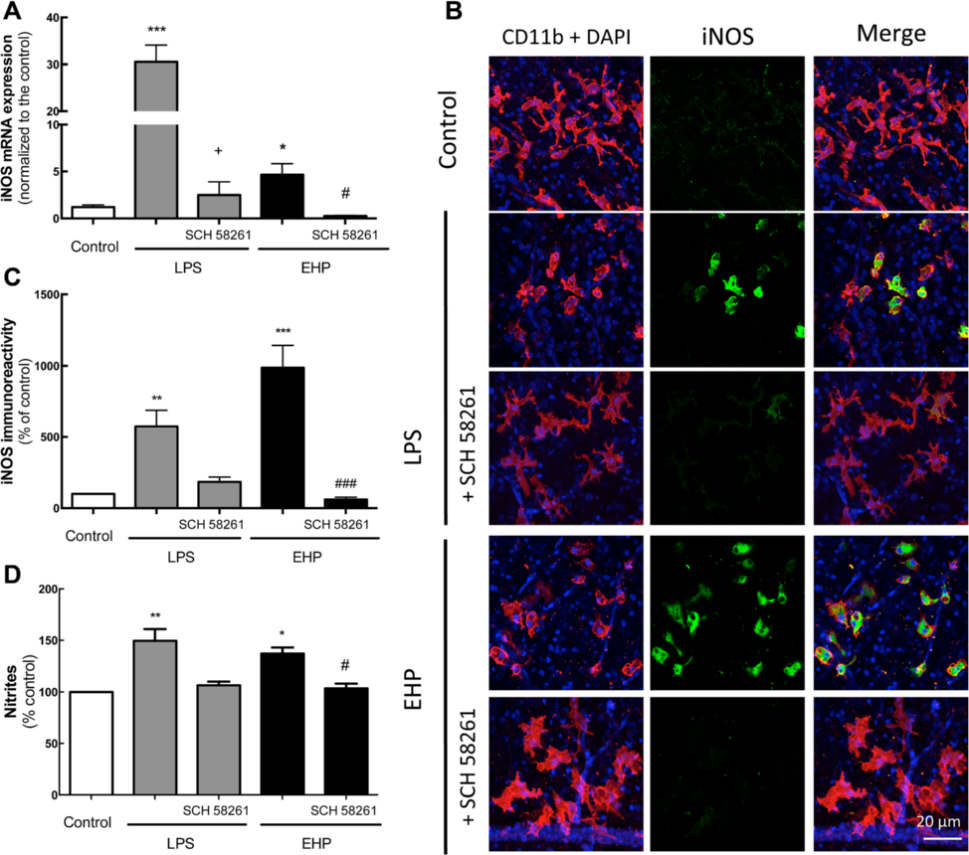
Blockade of A_2A_R decreases the expression and immunoreactivity of iNOS and NO production induced by LPS or EHP. Retinal organotypic cultures were pretreated with SCH 58261 (50 nM) and then challenged with LPS (3 μg/mL) or EHP (+70 mmHg) for 4 h. **a** iNOS mRNA expression was assessed by qPCR. Results are presented as fold change of the control, from six to twelve independent experiments. **b** Organotypic retinal cultures were immunostained for iNOS (*green*) and CD11b (microglia marker; *red*) and imaged in the GCL using a confocal microscope. Nuclei were stained with DAPI (*blue*). The images are representative of four to five independent experiments. **c** The immunoreactivity of iNOS in microglia localized in the GCL was quantified. Results are expressed as percentage of control from four to five independent experiments. **d** The production of NO was assessed by the Griess reaction in the culture supernatants, and nitrite formation was quantified. Results are expressed as percentage of control and are mean ± SEM of four to six independent experiments. *P < 0.05, **P < 0.01 and ***P < 0.001, different from control; ^+^ *P* < 0.05, different from LPS; ^#^ *P* < 0.05 and ^###^ *P* < 0.001, different from EHP; Kruskal-Wallis test, followed by Dunn’s multiple comparison test

As expected, the mRNA expression of iNOS significantly increased by 30.5-fold after 4 h of exposure to LPS (*n* = 6), and this effect was significantly decreased upon A_2A_R blockade (*n* = 4) (Fig. 3a). The exposure to EHP for 4 h also significantly increased iNOS mRNA expression by 4.6-fold over control (*n* = 5), and the blockade of A_2A_R also significantly prevented this effect (*n* = 6) (Fig. 3a).

In control conditions, the immunoreactivity of iNOS was barely detected in microglia localized in the GCL (Fig. 3b). Exposure to LPS or EHP for 24 h significantly increased iNOS immunoreactivity mainly in CD11b-immunoreactive cells (Fig. 3b, c), confirming that microglial cells are the main producers of NO under these conditions. This effect was abolished by blockade of A_2A_R, since iNOS immunoreactivity was similar to control (Fig. 3b, c).

The release of NO was indirectly quantified in the culture medium by Griess reaction 24 h after exposure to LPS or EHP (Fig. 3d). In control conditions, nitrite concentration was 5.64 ± 0.17 μM (*n* = 6). LPS or EHP significantly increased nitrite concentration to 149.5 ± 11 and 138 ± 3.5 % of the control, respectively (*n* = 4–5), and these effects were prevented by A_2A_R blockade (*n* = 3–4) (Fig. 3d).

#### A_2A_R blockade mitigated the inflammatory response induced by LPS or EHP

**Fig. 4:**
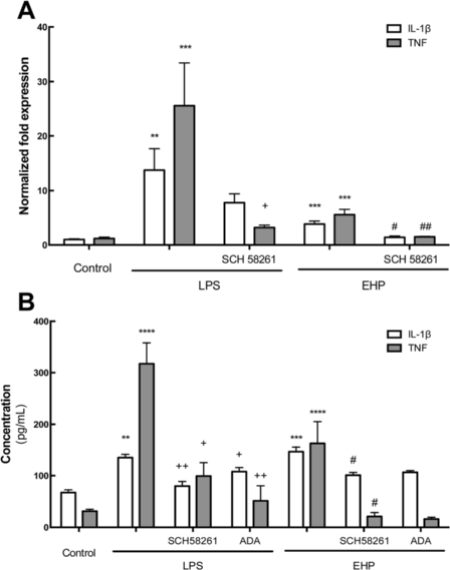
A_2A_R blockade partially inhibits the inflammatory response induced by LPS or EHP. Retinal organotypic cultures were pretreated with SCH 58261 (50 nM) and then challenged with LPS (3 μg/mL) or EHP (+70 mmHg) for 4 h. **a** Effects of A_2A_R blockade in the LPS- or EHP-induced mRNA expression of pro-inflammatory cytokines IL-1β and TNF were assessed by qPCR. Results are presented as fold change of the control, from six to thirteen independent experiments. **b** The release of IL-1β and TNF to the culture medium was quantified by ELISA. To evaluate the role of endogenous adenosine, the cultures were pretreated with adenosine deaminase (ADA; 1 U/mL). Results are expressed in picogrammes per milliliter and are mean ± SEM of five to ten independent experiments. **P < 0.01, ***P < 0.001 and ****P < 0.0001, different from control; ^+^ *P* < 0.05, and ^++^ *P* < 0.01, different from LPS; ^#^ *P* < 0.05 and ^##^ *P* < 0.01, different from EHP; Kruskal-Wallis test, followed by Dunn’s multiple comparison test

Activation of microglia leads to an increased expression and release of pro-inflammatory cytokines, such as IL-1β and TNF [19]. To further test if A_2A_R blockade prevented the LPS- and EHP-induced inflammatory response, we quantified mRNA levels encoding for IL-1β and TNF by qPCR. As shown in Fig. 4a, the exposure of retinal organotypic cultures to LPS or EHP for 4 h significantly increased the transcript levels of IL-1β and TNF (*n* = 5). Overall, the blockade of A_2A_R inhibited the LPS- and EHP-induced increase of IL-1β and TNF mRNA levels (*n* = 5–7) (Fig. 4a).

We next quantified the levels of IL-1β and TNF in the culture medium by ELISA (Fig. 4b). In control conditions, the concentration of IL-1β in the culture medium was 67.1 ± 5.5 pg/mL and the concentration of TNF was 30.9 ± 53.7 pg/mL (*n* = 10–15). Incubation with LPS or EHP for 4 h significantly increased IL-1β concentration in the culture medium to 135.3 ± 6.9 and 146.7 ± 9 pg/mL, respectively (*n* = 6–8), and the TNF concentration to 317.6 ± 40.6 and 162.8 ± 42.6 pg/mL, respectively (*n* = 6–9) (Fig. 4b). The blockade of A_2A_R significantly inhibited the LPS- and the EHP-induced increase of IL-1β or TNF levels in the culture medium (*n* = 5) (Fig. 4b).

Additionally, we tested if the removal of endogenous extracellular adenosine was equivalent to blocking A_2A_R in the control of LPS- or EHP-induced neuroinflammation. We found that the pretreatment of organotypic cultures with ADA (1 U/mL), which removes extracellular adenosine, abrogated the LPS- and EHP-induced increase in the expression (Fig. 4a) and extracellular levels (Fig. 4b) of both TNF and IL-1β (*n* = 3).

#### A_2A_R blockade prevented RGC death through the control of neuroinflammation

The elevation of the hydrostatic pressure is an experimental strategy to mimic in a retina culture model a situation of IOP increase, which is a major risk factor for glaucoma [2]. Studies from Sappington et al. [27] have already described RGC death under EHP conditions.

**Fig. 5:**
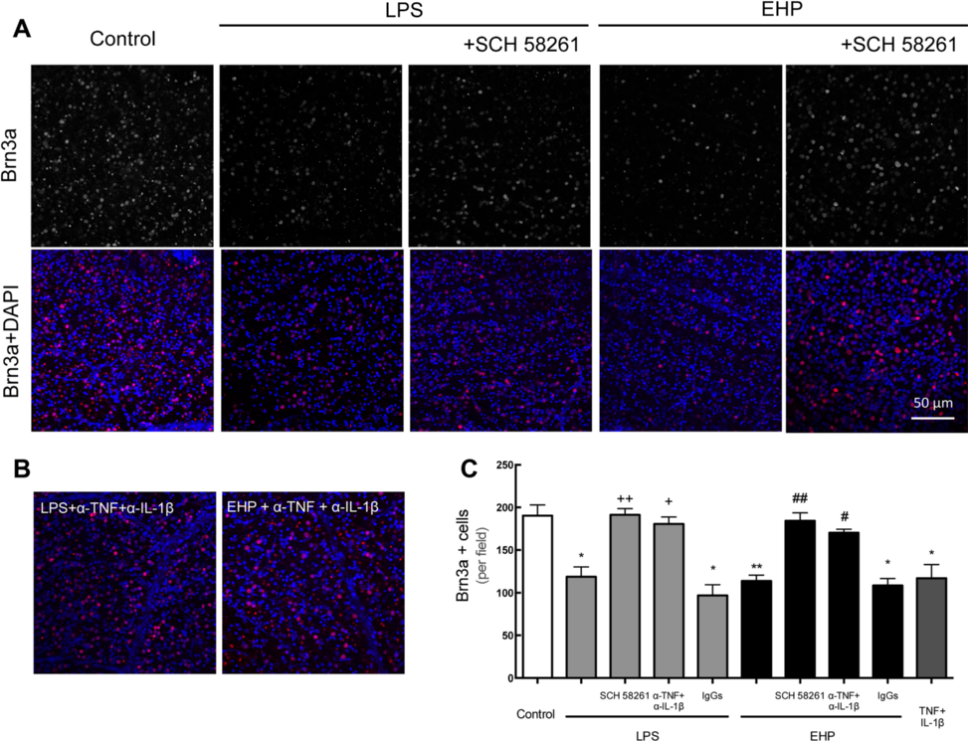
Blockade of A_2A_R and of TNF and IL-1 β prevents RGC death induced by LPS or EHP. Retinal organotypic cultures were pretreated with SCH 58261 (50 nM) or with anti-TNF and anti-IL-1β neutralizing antibodies and then challenged with LPS (3 μg/mL) or EHP (+70 mmHg) for 24 h. Rabbit and goat IgGs were used as control for the neutralization experiments. **a** Organotypic retinal cultures were immunostained for Brn3a (RGC marker, *red*) after treatment with SCH 58261 prior to challenge. Nuclei were stained with DAPI (*blue*). **b** Immunostaining with Brn3a (*red*) after treatment with neutralizing antibodies prior to challenge. Nuclei were stained with DAPI (*blue*). Representative images are depicted. **c** Surviving RGCs are presented as the number of Brn3a-immunoreactive cells per field and are mean ± SEM of five to seven independent experiments. *P < 0.05 and **P < 0.01, different from control; ^+^ *P* < 0.01 and ^++^ *P* < 0.01, different from LPS; ^#^ *P* < 0.05 and ^##^ *P* < 0.01, different from EHP; Kruskal-Wallis test, followed by Dunn’s multiple comparison test

Since A_2A_R blockade prevented microglia activation and the expression and release of pro-inflammatory cytokines, we next tested if A_2A_R blockade also prevented the loss of RGC induced by LPS or EHP in retinal organotypic cultures. Loss of RGCs was evaluated by counting the number of RGC, identified with an antibody against Brn3a (Fig. 5a), a marker of RGCs [39, 40]. The number of Brn3a-immunoreactive cells (Fig. 5a, c) significantly decreased when the retinal explants were exposed to LPS or EHP for 24 h, when compared with the control (190.5 ± 12 Brn3a-immunoreactive cells per field in control vs. 118.9 ± 11 and 113.9 ± 6 Brn3a-immunoreactive cells per field in LPS and EHP conditions, respectively, *n* = 6–7), indicating that both insults cause RGC loss. This effect was prevented with the treatment with A_2A_R antagonist (191.2 ± 7.3 and 184.3 ± 9.3 cells per field, respectively; Fig. 5a, c; *n* = 4–5).

Since A_2A_R blockade prevented both inflammatory responses and RGC loss triggered by LPS and EHP, we next investigated if TNF and IL-1β were necessary and sufficient to induce RGC loss under noxious conditions (LPS or EHP). Organotypic retinal cultures were pretreated with antibodies against TNF and IL-1β before incubation with LPS or exposure to EHP, in order to reduce the levels of both pro-inflammatory cytokines. The incubation of organotypic retinal cultures with antibodies against TNF and IL-1β prior incubation with LPS or exposure to EHP fully prevented the loss of RGCs (180.6 ± 8 and 170.2 ± 4 Brn3a-immunoreactive cells, respectively, *n* = 5; Fig. 5b, c). As a control, the incubation with rabbit and goat IgGs did not significantly inhibit the decrease in the number of RGC upon exposure to LPS or EHP (*n* = 4). In addition, incubation with TNF (20 ng/mL) plus IL-1β (10 ng/mL) was sufficient to induce loss of RGC (*n* = 3) (Fig. 5c) to an extent similar to that triggered by LPS or EHP. Moreover, incubation with the neutralizing antibodies in control conditions did not alter the number of RGCs present in the culture (data not shown). The neutralizing experiments under noxious conditions (LPS or EHP) fully recapitulated the incubation with SCH 58261, further supporting our conclusion that A_2A_R blockade control RGC loss through a control of retinal neuroinflammation.

## Discussion

The present work demonstrates that the blockade of A_2A_R prevented retinal neuroinflammation and death of RGC in an ex vivo model of glaucoma. We exposed retinal organotypic cultures to LPS and EHP, which bolstered microglia reactivity, increased neuroinflammatory response and loss of RGCs. These two noxious conditions up-regulated the A_2A_R system, as typified by an increase in the extracellular levels of ATP and increased expression and density of A_2A_R in microglia. Concomitantly, the A_2A_R system critically contributed to the neuroinflammation and RGC death, since A_2A_R blockade prevented the activation of microglia, the production of pro-inflammatory cytokines and the death of RGCs.

We took advantage of retinal organotypic cultures, a suitable model to evaluate cellular and molecular signalling mechanisms in which retinal anatomy is maintained [26] and which has been established as a convenient model for screening potential neuroprotective drugs in the retina [41]. This in vitro system enabled us to demonstrate that EHP changed microglia morphology towards an amoeboid-like form, similar to that caused by LPS, which has been extensively used as a microglial activator. Activation of microglial cells is observed as an early event in animal models of glaucoma [9, 42], in which increased IOP is a main risk factor [2]. In retinal organotypic cultures, the observed EHP- and LPS-induced microglia reactivity was paralleled by an increased expression and release of the pro-inflammatory cytokines IL-1β and TNF. Likewise, an increased production of TNF [11, 43] and IL-1β [44, 45] has been observed in glaucomatous animal models and in human glaucoma. Furthermore, the ability of anti-IL-1β and anti-TNF antibodies to prevent EHP-induced RGC death provided critical evidence that the death of RGCs upon exposure to EHP or LPS in retinal organotypic cultures actually resulted from the impact of pro-inflammatory cytokines. This is in agreement with previous reports demonstrating that the control of microglia reactivity [15–17] or of pro-inflammatory cytokines [4, 18, 46] prevents the loss of RGC in animal models of glaucoma. Nevertheless, the release of IL-6 by astrocytes and microglia triggered by EHP was reported to protect RGCs [27], although the authors used purified cultures of microglia, astrocytes and RGCs and did not evaluate the possible interactions between these cells in a more complex in vitro model, as the retinal organotypic culture. The globally deleterious role of microglia-associated pro-inflammatory status is further heralded by the report that minocycline, an inhibitor of microglia activation, reduced microglia activation and improved RGC axonal transport and integrity [15]. Overall, this evidence indicates that microglia reactivity is a precocious event and contributes to the pathophysiology of glaucoma by impairing the viability of RGCs.

The main conclusion of this study was the critical role of A_2A_R in the control of EHP- or LPS-induced microglia activation, production of pro-inflammatory cytokines and RGC death in retinal organotypic cultures. Indeed, we observed that the blockade of A_2A_R prevented the EHP- or LPS-induced modification of the production of pro-inflammatory cytokines and of NO as it was previously observed in the rodent hippocampus [24]. Accordingly, it was already demonstrated that activation of A_2A_R potentiates NO release from reactive microglia in culture, an effect that was associated with microglia neurotoxicity, and A_2A_R antagonist was suggested as a potential neuroprotective drug [22]. Moreover, we observed that A_2A_R blockade prevents EHP-induced microglia morphological alterations, in agreement with recent findings that A_2A_R antagonism reduces the retraction of processes in LPS-activated microglia [47].

These conclusions seems to contradict previous studies reporting that the activation of A_2A_R reduces microglia reactivity using primary retinal microglia cultures exposed either to LPS, hypoxia or amadori-glycated albumin [48–50]. Several factors may explain this discrepancy: (1) while others used cultures of microglial cells, we used an organotypic retinal culture in which all retinal cells are present, and thus, an additional contribution from other glial cells cannot be excluded [51, 52]; this is particularly important given that the control by A_2A_R of microglia reactivity can be shifted from inhibitory to excitatory by the presence of increased extracellular levels of glutamate [53]; (2) the insults triggering microglia activation are different and the LPS concentrations and time points were different; and (3) CGS 21680, the A_2A_R agonist, at the concentration used in those studies (20 and 40 μM) is no longer selective, being proposed to bind also to A_1_R [54, 55]. The A_1_R is coupled to G_i/o_-proteins and often inhibitory, whereas the A_2A_R is usually coupled to G_s_-proteins, enhancing cAMP accumulation and PKA activity [56]. Nevertheless, the observation of different responses in different models should be taken into account due to the dual role of adenosine receptors and different responses of microglia, which can be elicited with different stimuli and environmental conditions [57]. In fact, in the brain, it is the blockade rather than the activation of A_2A_R than reduce microglia activation and neuroinflammation upon different noxious stimuli [58, 24]. This probably contributes to the neuroprotection afforded by A_2A_R antagonists in brain diseases with a neuroinflammatory involvement such as ischemia, epilepsy, traumatic brain injury, multiple sclerosis or Alzheimer’s or Parkinson’s disease (reviewed in [25]). Accordingly, we also observed that A_2A_R blockade prevented the LPS- and the EHP-induced RGC death in retina organotypic cultures. This might result from the ability of A_2A_R to control the activation of microglia and the production of pro-inflammatory cytokines that we showed to be sufficient and necessary to trigger RGC death, but it may also involve an ability of A_2A_R to directly control neuronal viability. In fact, neuronal A_2A_R can directly affect the degeneration of mature neurons upon exposure to different stimuli (e.g. [59, 60]), namely to pro-inflammatory cytokines [61], whereas they have an opposite effect in immature neurons [62, 63] and during neurodevelopment [64].

In our work, the relevance of the A_2A_R modulation system in the control of RGC death through a control of neuroinflammation in the retina is further underscored by the observed up-regulation of this system in retinal organotypic cultures exposed either to LPS or to EHP. In fact, LPS and EHP caused an increase in the extracellular levels of ATP. The cellular source of this extracellular ATP is not clear, but it can be released from different cells in the retina, such as RGCs [65], microglia [66] and Müller cells [67]. Moreover, recent work demonstrated that astrocytes present in the optic nerve head can also release ATP through pannexin channels in response to a mechanical strain, suggesting this mechanism as a source of extracellular ATP under chronic mechanical strain, as occurs in glaucoma [68]. Actually, elevated levels of extracellular ATP have been reported in the retina as a response to an acute rise in ocular pressure [69, 70], and the ATP levels are elevated in the aqueous humour of patients with primary acute and chronic angle closure glaucoma, which presents evidence for a contribution of the purinergic signaling in this disease [71, 72]. The increased levels of ATP can function as a danger signal [73] and can either activate P2 receptors, namely P2X7 receptors in the retina [74, 65, 75, 76], or be extracellular catabolized by ecto-nucleotidases into extracellular adenosine that preferentially activates A_2A_R [77, 36]. Remarkably, EHP and LPS not only bolstered the source of adenosine activating A_2A_R but also triggered an increased expression of A_2A_R, which was translated into an increased density of A_2A_R in microglia. This is in accordance with the up-regulation of A_2A_R that is observed upon different noxious conditions (reviewed in [78, 25]), namely in microglia [79, 24, 80]. Thus, noxious stimuli such as LPS or EHP triggered an up-regulation of the A_2A_R system in retinal microglia, which critically contributes to the development of neuroinflammation and RGC death. We cannot rule out the role of A_2A_R present in other cell types of the retinal organotypic culture, but in the GCL, A_2A_R was found to be mainly located in microglia. Furthermore, additional studies will be required to determine if A_2A_R blockade only affords a prophylactic benefit or may also be therapeutically effective.

## Conclusions

The present results demonstrate that EHP can lead to an inflammatory response, similar to LPS, which is associated with the death of RGC. Thus, the organotypic retinal culture exposed to EHP may be an important experimental model to investigate neuroprotective and anti-inflammatory pharmacological strategies against RGC death. Herein, we demonstrate for the first time that A_2A_R blockade prevents retinal microglia reactivity and pro-inflammatory responses triggered by LPS or EHP and confers neuroprotection to RGC by controlling retinal neuroinflammation induced by EHP or LPS. This prompts the hypothesis that A_2A_R antagonists might have therapeutic potential in the treatment of glaucoma, a proposal re-enforced by the report that IOP could be reduced by a selective A_2A_R antagonist, ZM 241385, in mice [81].

## Abbreviations

ADA: Adenosine deaminase
ATP: Adenosine triphosphate
A_2A_R: A_2A_ receptor
CTCF: Corrected total cell fluorescence
EHP: Elevated hydrostatic pressure
GCL: Ganglion cell layer
IL: Interleukin
iNOS: Inducible nitric oxide synthase
IOP: Intraocular pressure
LPS: Lipopolysaccharide
NO: Nitric oxide
PBS: Phosphate-buffered saline
RGC: Retinal ganglion cell
TNF: Tumour necrosis factor
